# From Journals to Bedside: We Must Improve the Compliance with Practice Guidelines

**DOI:** 10.5935/abc.20180186

**Published:** 2018-10

**Authors:** Barbara Kumagai, Bruno Caramelli

**Affiliations:** Unidade de Medicina Interdisciplinar em Cardiologia, InCor, HCFMUSP, São Paulo, SP - Brazil

**Keywords:** Practice Guidelines as Topic, Myocardial Infarction, Perioperative Care, Platelet Aggregation Inhibitors/therapeutic use, Medication Adherence, Treatment Refusal

In this issue of “Arquivos Brasileiros de Cardiologia”, Borges et al.^1^ described the rate of non-compliance with practice guidelines in a Hospital-based study regarding the use of antiplatelet agents in the perioperative setting of non-cardiac surgery.^1^ The authors found an extremely high non-compliance rate of 80.75%, and depicted a significant negative association among non-compliance, patient education level, and the presence of previous myocardial infarction. The authors concluded that local procedures and protocols must be urgently defined.

Perioperative care underwent profound changes in the past decades. Initially, evaluation was limited to issues related to the anesthetic procedures, or to the cancellation of interventions for patients at high risk of complications. Eventually, population aging, the improvement in surgical techniques, and the development of less invasive procedures brought to operation theaters patients at an increased risk of complications, especially cardiovascular ones. Perioperative care specialists had to develop new and interdisciplinary skills to deal with several aspects of medicine, kindly deserving the nickname *Chameleon doctor*.^2^^-^^4^

Among perioperative complications, cardiovascular are the most feared and strongly related to mortality and morbidity. Myocardial infarction complicating non-cardiac surgery represents a big challenge, especially after the elegant demonstration that almost half of the events involves coronary thrombosis in the pathophysiology, and are not a simple consequence of increased oxygen demand or decreased supply.^5^ This latter issue, in a scenario of an increasing number of coronary Stenting procedures, requires recommendations for physicians working at the point-of-care. Elaborated by experts and frequently supported by medical associations, practice guidelines serve also as a reference for public and private health systems approval and reimbursement.^6^ Previous authors have also found elevated non-compliance rates in different areas of medicine both at local and country level. However, the non-compliance rate regarding the management of antiplatelet agents in the perioperative setting, has not been previously studied. Despite analyzing a small sample size and one Hospital, the study by Borges et al. is very welcome, and stands out because of the astonishing high non-compliance rate of more than 80%.

At a closer look, however, two other aspects came out and must be highlighted:

1. **Treatment delivered without evidence-based support**
  The most worrying aspect is the finding that almost 30% of the patients were taking antiplatelet agents for primary prevention of cardiovascular diseases. Unfortunately, this treatment is not fully supported by clinical data, even for patients at high cardiovascular risk.
2. **Underrepresentation of some surgical specialties**
  According to clinical practice guidelines, there are only two specific conditions where antiplatelet agents are not safe and must be suspended before non-cardiac surgery: intracranial and transurethral resection of the prostate because of the limited possibility for local compression in order to stop bleeding. In Borges et al.’s study, however, urological interventions represent only 6.8% of the group, and neurological interventions were not included. This finding leads us to conclude that observed interruptions (or not) of the antiplatelet agent refers, most of the times in the present study, to their use as a primary prevention drug.
3. **Is it correct to consider some aspects related to the use of non-evidence-based treatment as non-compliance?**
  Taking in account aspects 1 and 2 above, one can depict that, indeed, most patients in the present study did not interrupt or incorrectly interrupt the antiplatelet drug that was incorrectly prescribed (18.6 + 26.1 + 13 = 57.7% on Table 2). Despite the importance of the finding in Borges et al.’s study, we think that their results could be contained in two major findings:
  - Antiplatelet agents are frequently overprescribed, and this issue can have consequences for patients that may be submitted to surgery in the future.
  - Interrupting an antiplatelet agent, going against practical guidelines recommendations, is frequent and can have consequences for patients at an increased cardiovascular risk in the perioperative period.

In conclusion, the interesting study by Borges et al. tells us that training is urgently needed to improve perioperative care and cardiovascular primary prevention.
